# Determinants of Physiological and Perceived Physiological Stress Reactivity in Children and Adolescents

**DOI:** 10.1371/journal.pone.0061724

**Published:** 2013-04-19

**Authors:** Brittany E. Evans, Kirstin Greaves-Lord, Anja S. Euser, Joke H. M. Tulen, Ingmar H. A. Franken, Anja C. Huizink

**Affiliations:** 1 Department of Child and Adolescent Psychiatry/Psychology, Erasmus University Medical Center/Sophia Children's Hospital, Rotterdam, The Netherlands; 2 Department of Developmental Psychology and the EMGO Institute for Health and Care, VU University Amsterdam, Amsterdam, The Netherlands; 3 Department of Psychology, Erasmus University Rotterdam, Rotterdam, The Netherlands; 4 Department of Psychiatry, Erasmus University Medical Center, Rotterdam, The Netherlands; Scientific Directorate, Bambino Hospital, Italy

## Abstract

**Aims:**

Abnormal physiological stress reactivity is increasingly investigated as a vulnerability marker for various physical and psychological health problems. However, studies are inconsistent in taking into account potential covariates that may influence the developing stress system. We systematically tested determinants (individual, developmental, environmental and substance use-related) of physiological and perceived physiological stress reactivity. We also examined the relation between physiological and perceived physiological stress reactivity.

**Method:**

In a stratified sample of 363 children (7–12 years) and 344 adolescents (13–20 years) from the general population, we examined cortisol, heart rate, respiratory sinus arrhythmia and perceived physiological stress reactivity to a psychosocial stress procedure.

**Results:**

Using multivariate linear regression models, we found that individual, developmental, environmental and substance use-related factors were related to each of the stress response indices. These determinant factors were different for each of the stress reactivity indices, and different in children versus adolescents. Perceived physiological stress reactivity predicted cortisol reactivity in adolescents only. All other relations between perceived physiological and physiological stress reactivity were not significant.

**Conclusions:**

As physiological stress variables are often examined as vulnerability markers for the development of health problems, we maintain that it is essential that future studies take into consideration factors that may account for found relations. Our study provides an overview and indication of which variables should be considered in the investigation of the relation between physiological stress indices and illness.

## Introduction

Abnormal physiological stress reactivity is increasingly investigated as a potential vulnerability marker for various physical and psychological health problems, such as cardiovascular diseases [1], anxiety and depressive disorders [2], [3] and disruptive behavioral disorders [4] to name only a few. However, studies vary considerably as to which variables are included as covariates; as yet there is no consensus on this matter. A number of theories (i.e. [5], [6], [7]) do outline several proposed determinants of physiological stress reactivity, which are individual, developmental, environmental and substance use-related in nature. Determinants of the physiological stress response have been extensively reviewed, especially concerning hypothalamic-pituitary-adrenal (HPA) axis responses (i.e. [8], [9], [10], [11]). However, few studies have systematically *tested* such determinants at once. The first aim of this paper was therefore to address this gap in the literature by testing a large number of potential determinants of stress reactivity.

A second concern arises from the physiological stress literature. Physiological stress reactivity is frequently considered a single construct, often indexed by only one measure of physiological stress (i.e., heart rate). Yet, different indices of physiological stress do not always follow the same response pattern [12]. Moreover, it is not known whether the same factors can be considered determinants of the different physiological stress indices. Therefore, our study tested potential determinants of cortisol, heart rate, respiratory sinus arrhythmia (RSA) and perceived physiological stress (PPS; i.e. the subjective impression of physiological stress) reactivity. On a similar note, physiological stress is generally postulated to serve as an index for PPS [13], yet, convincing empirical evidence for this is limited (i.e. [14]). The second aim of this study was to examine the extent to which PPS could predict physiological stress reactivity.

A third concern is that the majority of the literature concerning physiological stress determinants has focused on adults. In children and adolescents, very little is known about which covariates should be taken into account, despite a myriad of studies investigating physiological stress as a vulnerability factor for disorders in childhood and adolescence (i.e. [15]). This transitional period from childhood to adolescence is particularly relevant as development is ongoing, and research suggests that stress responses are developing and may not be uniform during this period [16], [17], [18]. One study investigated determinants of the cortisol response in 10–12 year-olds [19], and we intend to extend this study by 1) examining not only individual and developmental determinants, but also environmental and substance use-related, 2) by examining these determinants in relation to heart rate, RSA and PPS as well as cortisol reactivity, and 3) by performing the study in children as well as adolescents with a wider range of ages.

Two main systems make up the physiological stress response: the autonomic nervous system and the HPA axis. The autonomic nervous system is comprised of the parasympathetic nervous system and the sympathetic nervous system. According to polyvagal theory, the ventral branch of the vagus (the primary component of the parasympathetic nervous system) is responsible for maintaining homeostasis during rest, thereby keeping heart rate low [20], [21]. When an organism is confronted with a stressor, the most immediate response involves vagal withdrawal, which leads to an increase in heart rate, indicating the organism's preparedness to respond to an anticipated stressor. If this response is insufficient, the phylogenetically older sympathetic nervous system is activated, entailing the fight-or-flight response which elevates heart rate (further). RSA is frequently assessed and is considered a valid index of vagal tone [20].

The HPA response to stress entails the production of corticotropin-releasing hormone by neurons in the paraventricular nucleus of the hypothalamus. This stimulates the secretion of adrenocorticotrophic hormone in the pituitary which in turn stimulates the secretion of cortisol in the outer cortex of the adrenal gland. As the cortisol in saliva is unbound and biologically active [22], salivary cortisol is often used because of its methodological facileness for participants. When confronted with a stressful situation, the adaptive response of a healthy individual is a temporary increase in the secretion of cortisol (e.g. [23]), which occurs approximately 20 minutes subsequent to the onset of the stressor [24]. The HPA stress response is influenced by endogenous and exogenous stressors, including psychological stress [25], [26], which is of interest here because of its ecological validity.

The Trier Social Stress Task (TSST; [27]) is a valid and widely used task to induce physiological stress responses [28]. Participants are asked to perform a mental arithmetic task and a personal speech in front of judges and/or a camera, thereby provoking psychosocial stress. The most important elements of the task are uncontrollability and social-evaluative threat [29]. The task used in the present study was modeled closely after the TSST.

### Determinants of the stress response

Determinants of stress reactivity were outlined in previous theoretical models (i.e. [5], [6], [7]), and reviews (i.e. [8], [9], [10], [11]). Below such factors are introduced; a review is beyond the scope of this work.

Individual differences have quite frequently been investigated in previous studies, most often in relation to cortisol reactivity. For example, *sex* differences are often reported in adults (e.g. [30]), although in younger subjects the results are equivocal (i.e. [31], [32], [33], [34]). The *menstrual cycle phase* and *oral contraceptive use* of females may account for reported sex differences, as has been found in adults ([35], [36], [37], but see also [38], [39]), yet this finding was not replicated in a sample of adolescents [34]. Other factors such as *ethnicity* [40], *body mass index* (BMI; [32], [40], [41], [42]) and low *birth weight* (e.g. [43], [44], [45]) have similarly been found to affect physiological stress reactivity. *Temperament* is defined as a set of inherited personality traits that are observable early in life [46], and is theoretically postulated to be closely related to physiological stress (e.g. [46], [47], [48]). Empirical investigations have confirmed this in young children [49], [50], but it is unclear whether such effects continue to be of importance during adolescence.

Developmental differences in physiological stress reactivity are especially relevant in children and adolescents [16], [51]. Stress reactivity patterns differ between childhood and early adulthood [18], [33], [52]. Because pubertal changes mark the transition into adolescence [53], it is interesting to examine whether *age* effects are independent of *pubertal stage*, as a convincing confirmation of this hypothesis is lacking (see [32], [33], [52], [54]).

Early environmental adversity has been related to subsequent physiological hyper-reactivity (e.g. [55]) and hypo-reactivity (e.g. [56], [57]). Previous studies have found various indices of environmental adversity to influence stress reactivity, such as *socioeconomic status* (*SES*; [40], but see also [58]), *urbanicity* (i.e. how urban the area one lives in is; [59], *family situation* (i.e. one- or two-parent household; [60]), *parenting behaviors* [55], [61], [62], and *adverse life events* [40], [57], [63], [64], [65], [66].

Lifestyle-related factors, such as substance use, are also proposed to influence physiological stress reactivity. For instance, researchers have reported an influence of *caffeine* [10], *tobacco*, *alcohol* [11] and *cannabis use* [67] on physiological stress reactivity.

In sum, before firm conclusions can be drawn on the validity of stress reactivity as a vulnerability marker for (mental) health problems during childhood and adolescence, we must clarify how stress measures relate to each other, and how they are influenced by determinants. The first aim of this study was to examine to what extent individual, developmental, environmental and substance use-related factors are related to physiological (cortisol, heart rate and RSA) and PPS reactivity. We examined children and adolescents separately because some determinants (i.e. parenting behaviors) may influence children and adolescents differentially, and others (i.e. alcohol use) are only appropriate for adolescents. For both children and adolescents, we investigated the individual determinants *sex*, *ethnicity*, *BMI*, *birth weight*, and temperament characteristics *shyness*, *activity*, *emotionality* and *sociability*; the developmental determinant *age*; the environmental determinants *urbanicity*, *SES*, *family situation*, and parenting behaviors *emotional warmth*, *overprotection*, *rejection*, *involvement*, *positive parenting*, *poor monitoring*, *inconsistent discipline* and *corporal punishment*, and *adverse life events*; and *cola use*. In children only, we additionally investigated *menstruation*, as a proxy of pubertal development in girls. In adolescents only, we additionally investigated the individual determinants *menstrual cycle phase* and *oral contraceptive use* in girls; developmental determinant *pubertal stage*; and *coffee*, *tobacco*, *alcohol* and *cannabis use*. The second aim of this study was to examine the relation between PPS and physiological stress reactivity.

## Methods

### Participants

**Figure 1:**
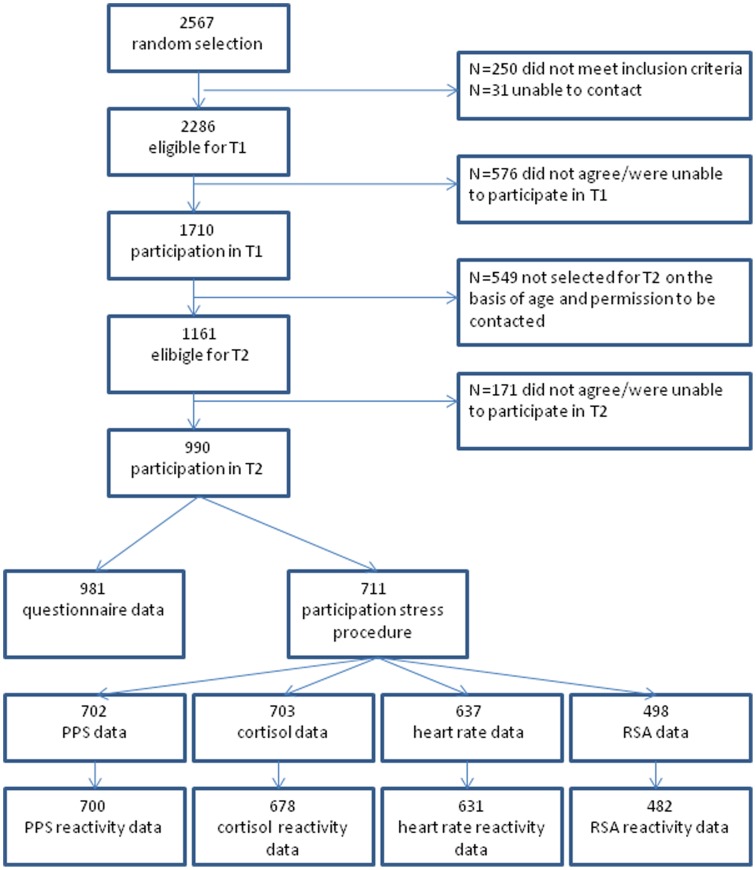
Flow-chart of available data. *Notes.* PPS = perceived physiological stress; RSA = respiratory sinus arrhythmia.

Participants in this study are part of an ongoing longitudinal general population study [68]. At the first assessment wave (T1), 2286 eligible children and adolescents were randomly drawn from the registers of 35 municipalities in the Dutch province of South Holland. Of these, 1710 individuals participated in T1. At T2, 1161 of the participants fulfilled inclusion criteria, of whom 990 participated. The T2 measurement consisted of questionnaires as well as a psychosocial stress procedure, which took place between November 2004 and March 2009. Youth (*n* = 711) between the ages of 7 and 20 years participated in this stress procedure, for whom complete data on at least one of the stress reactivity variables was available for 707 individuals (47% boys, average age 13.77 years, SD 3.56). All measures reported on in this study were obtained at T2, with the exception of SES, which was assessed at T1. As compared to the sample of 1161 eligible participants for T2, female sex (*p*<.05; R^2^ = .01), younger age (*p*<.01; R^2^ = .01), and a higher SES (*p*<.001; R^2^ = .02) predicted inclusion in the current sample of 707. Participants and non-participants did not differ on behavioral problems or anxious/depressive symptomology at T1 or T2. See Figure 1 for a flow chart of available data. Written informed consent was obtained from all participants and their parents and participants received a gift certificate. The Erasmus University Medical Center (Erasmus MC) Ethics Committee approved of the study.

### Psychosocial stress procedure

Stress procedure sessions began at 12 pm or at 3 pm in order to minimize differences due to diurnal variation of cortisol levels. Stress procedure sessions commenced with an explanation of the procedure by the experiment leader. After the completion of a questionnaire set, the electrodes of the electrocardiogram were attached and participants were told to breathe normally and to relax. After a ten minute pre-task rest period, the social stress tasks began, which were characterized by uncontrollability and social-evaluative threat, thus designed to elicit a stress reaction [29]. These tasks entailed a mental arithmetic task (i.e. incremental subtraction; 4 min), a public speaking task (imagine that one was accused of stealing from the school/workplace cafeteria, response in front of the experiment leader and a video camera; 8 min mental preparation, 6 min speech) and a computer mathematics task (mentally ordering numbers; 5 min; see [69] for full details on the procedure). The session ended with a five minute recovery period and a relaxing nature documentary (25 min). The maximum stress response was evoked by the mental arithmetic task in 23.6%–39.2% of individuals, by the public speaking task in 39.6%–55.4% of individuals, and by the computer mathematics task in 11.1%–58.7% of individuals for all stress responses.

### Perceived physiological stress

Self-reported perceived physiological stress (PPS; [69]) was assessed after the rest periods and after each of the tasks. Participants answered seven questions (e.g. ‘Can you feel your heart beating?’, ‘Are you nervous?’) using a visual thermometer ranging from 0 (not at all) to 8 (very much). The scores were summed to a total score of PPS for each period/task. The reliability and validity of this measure has not been examined formally, but has been used in several studies (i.e. [69], [70]). Cronbach's alphas for the scale at each period ranged between .63 and .81 for the entire sample.

### Physiological stress measures

#### Cortisol

After each period/task, at the middle of the documentary and at the end of it, the participant was asked to provide saliva samples. These samples reflect activity in the hypothalamus approximately 20 minutes earlier due to the delay in observable cortisol response [71]. Saliva samples were kept in a freezer at −20 degrees Celsius [72] and were collectively sent to the laboratory for analysis. A time-resolved fluorescence immunoassay was implemented to determine the cortisol concentration. Outliers greater than 3 SD above the mean were removed from the analysis due to possible contamination (e.g. blood, medicine).

#### Heart rate

Heart rate was measured using a three-lead electrocardiogram and monitored constantly throughout the entire stress procedure. The electrocardiogram was sampled at 512 Hz and stored on a flashcard by means of a portable digital recorder (Vitaport™ System; TEMEC Instruments B.V., Kerkrade, NL). After completion of the recording, physiological data were imported and processed on a Personal Computer using a Vitascore™ software module (TEMEC Instruments BV, Kerkrade, NL). A customized software program calculated the interbeat intervals of the electrocardiogram using R-top detection, resulting in interbeat intervals time series. This time series was inspected to detect and remove artifacts. Heart rate time series were calculated from these interbeat intervals and expressed in beats per minute (bpm); the heart rate time series were subsequently averaged per period during the stress procedure.

#### Respiratory sinus arrhythmia

In order to compute an index of RSA, the heart rate time series during the pre-task rest period, the preparation part of the public speaking task, the computer task and the post-task recovery period (i.e. those periods when speaking did not occur) were scrutinized for stationarity. The heart rate time series were subsequently subjected to a discrete Fourier transformation, based on non-equidistant sampling of the R-wave incidences (CARSPAN program, Groningen, The Netherlands; [73], [74]), to yield power spectra of the rhythmic oscillations over a frequency range of 0.02–0.50 Hz, with a resolution of 0.01 Hz. For each period, the power in the high frequency band (0.14–0.5 Hz) of the heart rate time series was calculated as an index of RSA. The data were natural log transformed in order to obtain normal distributions.

For heart rate and PPS, the reactivity measure consisted of the maximum averaged value during the three stressful tasks minus the averaged value of the pre-task rest period. For RSA, we subtracted the minimum averaged value during the tasks from that during the pre-task rest period. Cortisol reactivity was the difference between the maximum value corresponding to cortisol levels during the three stress tasks and the second pre-task value. The first cortisol pre-task value was excluded from the analyses as it was generally higher, thus most likely reflecting anticipatory stress to a greater degree than the second measurement. One extreme outlier in the heart rate difference score was excluded from the analysis.

### Determinants of the stress response

#### Individual and developmental factors

*Age*, *sex* and *ethnicity* were reported by the mother in a general demographics questionnaire. Ethnicity was coded as either of Dutch origin (x = 0) or not of Dutch origin (x = 1). We assessed *pubertal stage* using self-reported Tanner stages [75] in adolescents only. A Health Questionnaire, developed by Erasmus MC researchers specifically for this study, was used to inquire after general health issues and use of substances. The questionnaire included a parent as well as a self-report version. For all variables the self-report items were used for analysis, except in the cases of missing items, for which the parent report was consulted. In the child sample, *menstruation* (i.e. are you menstruating yet?, no: x = 0; yes: x = 1) was assessed as a proxy for pubertal development in girls. In the adolescent sample, for free-cycling girls who indicated having a regular cycle (N = 69), *menstrual cycle phase* was calculated based on the self-reported first day of the last menstrual cycle, coded as either follicular (0–14 days prior to the test session; x = 0) or luteal (15–35 days prior; x = 1). We also assessed *OC use* (no: x = 0; yes: x = 1) with this questionnaire. Prior to the test session, height and weight were measured in order to calculate *BMI*. The test assistant obtained the *birth weight* from a personal record of birth-related variables issued by the hospital upon birth and brought to the test session by the participant. Parent-reported temperament variables were based on the Emotionality, Activity, Sociability scale [76], which consists of 20 items scored on a scale of 1 (not at all) to 5 (very much) and contains four subscales: *emotionality* (i.e. ‘reacts intensely when upset’), *activity* (i.e. ‘is very energetic’), *sociability* (i.e. ‘likes to be with people’) and *shyness* (i.e. ‘takes a long time to warm up to strangers’). Mother and father reports were averaged. We excluded item 18 (i.e. ‘when alone, child feels isolated’) from the *sociability* scale in both the child and adolescent samples, and item 7 reversed (i.e. ‘when child moves about, he/she moves slowly’) from the *activity* scale in the adolescent sample in order to increase reliability. Please see Table 1 for the Cronbach's alphas of the subscales of all questionnaires.

**Table 1:** Descriptive statistics of all potential determinants of stress reactivity.

Sample	Children				Adolescents			
Variable	*N*	F (%) or Range	*M*(*SD*)	C α (m;f)	*N*	F (%) or Range	*M*(*SD*)	C α (m;f)
Sex (male/female)	363	49.9/50.1			344	43.9/56.1		
Ethnicity (Dutch/non-Dutch)	363	79.9/20.1			344	83.1/16.9		
Body mass index	355	13.16–31.99	18.29(3.04)		336	15.82–40.56	21.88(3.41)	
Percentiles (25/50/75)			16.1/17.8/19.8				19.6/21.1/23.5	
Birth weight (kilograms)	311	1.31–6.00	3.43(0.58)		258	1.26–5.00	3.34(0.57)	
Menstrual cycle phase (follicular/luteal)					67	49.3/50.7		
Oral contraceptive use (no use/use)					184	48.4/51.6		
Temperament: Shyness	262	5.00–22.00	11.14(3.19)	.74; .68	311	5.00–20.00	10.45(2.94)	.70; .73
Activity	262	9.00–23.50	15.91(2.91)	.63; .65	312	6.00–22.00	14.29(2.59)	.69; .60
Emotionality	263	5.00–23.00	12.32(3.44)	.76; .77	312	5.00–21.00	10.21(3.10)	.78; .79
Sociability	263	8.00–20.00	14.64(2.56)	.72; .69	312	5.00–20.00	14.33(2.69)	.76; .69
Age	363	7.92–12.92	10.62(1.40)		344	13.00–20.83	17.10(1.55)	
Pubertal stage					301	2.00–5.00	4.33(0.69)	
Menstruation (no/yes)	179	86.6/13.4						
Urbanicity (rural/town/urban)	363	12.1/57.3/30.6			344	14.5/54.9/30.5		
SES (low/average/high)	360	4.2/51.1/44.7			343	3.8/52.5/43.7		
Family situation (two/one parent)	261	88.9/11.1			300	89.0/11.0		
Parenting (EMBU-C): Emotional warmth	352	1.82–4.00	3.31(0.48)	.91; .91	324	1.53–4.00	3.10(0.50)	.90; .93
Overprotection	352	1.00–3.33	1.90(0.39)	.68; .65	323	1.00–2.88	1.81(0.34)	.71; .70
Rejection	352	1.00–2.53	1.45(0.29)	.80; .83	324	1.00–3.03	1.35(0.26)	.84; .86
Parenting (APQ): Involvement	263	2.35–4.80	3.77(0.41)	.73; .72	313	1.55–4.50	3.46(0.44)	.70; .78
Positive parenting	263	2.67–5.00	3.79(0.51)	.80; .76	313	1.75–4.83	3.48(0.49)	.74; .77
Inconsistent discipline	263	1.08–4.00	2.40(0.45)	.60; .64	311	1.00–3.50	2.23(0.52)	.74; .75
Corporal punishment	262	1.00–3.75	1.29(0.42)	.67; .63				
Adverse life events					344	0.00–9.00	2.57(1.94)	.54
Cola use (no use/use)	362	33.1/66.9			339	20.9/79.1		
Coffee use (no use/use)					339	67.6/32.4		
Tobacco use (never or little/every day)					318	85.8/14.2		
Alcohol use (no use/use)					342	13.7/86.3		
Cannabis use (no use/use)					315	75.9/24.1		

*Notes.* F = frequency in percentage; Cα = Cronbach's alpha; m = mother; f = father; SES = socioeconomic status.

#### Environmental factors

*Urbanicity* was based on the population rate of the reported home city/town of the participant at the time of the test session, coded as rural (x = 0), town (>10,000 inhabitants; x = 1) or urban (>100,000 inhabitants; x = 2; [77]). Population statistics were based on online national archives [78]. Mother-reported *SES* was based on the higher occupational level of either parent [79] and coded into low (x = 0), average (x = 1) and high (x = 2) *SES*. *Family situation* of the child was based on parent reports and categorized as two-parent household (x = 0) and one-parent household (x = 1). An ‘other’ category was available in the questionnaire, but participants who marked this were excluded from the relevant analyses.

Parenting style was assessed with a modified version of the EMBU-C, a child report version of the EMBU (Egna Minnen Beträffande Uppfostran; a Swedish acronym for My Memories of Upbringing; [80]), which measures the child's perception of his/her upbringing. The version we used is to a large extent in accordance with a shorter Dutch version of the EMBU [81] and contains 47 items in total that were answered on a four-point likert scale from 1 (no, never) to 4 (yes, most of the time). Three scales were derived: *emotional warmth* (i.e. ‘my mother/father accepted me as I was’, *rejection* (i.e. ‘it was difficult to approach my mother/father’) and *overprotection* (i.e. ‘I wished my mother/father would worry less about what I was doing’). For each EMBU-C item, participants assessed both the father's and the mother's parenting behaviors. Items for parents' parenting behaviors were averaged in order to achieve a general view of the parenting environment.

Parent-self-reported parenting practices were assessed using the Alabama Parenting Questionnaire (APQ; [82]). The questionnaire consists of 42 items and five subscales, namely *involvement* (i.e. ‘you ask your child about his/her day in school’), *positive parenting* (i.e. ‘you praise your child if he/she behaves well’), *poor monitoring* (i.e. ‘your child is out with friends you do not know’), *inconsistent discipline* (i.e. ‘you threaten to punish your child and then do not actually punish him/her’) and *corporal punishment* (i.e. ‘you slap your child when he/she has done something wrong’). Parents rated on a five-point likert scale ranging from 1 (never) to 5 (always) to what extent they displayed the described parenting behavior. For this study, mother and father reports were averaged. Due to insufficient internal reliability, we excluded the *poor monitoring* scale in both the child and adolescent samples, as well as the *corporal punishment* scale in the adolescent sample.

*Adverse life events* were selected from an extensive Life Events Questionnaire (LEQ; [83]) which included both severely and mildly adverse events as well as positive events, and from the post traumatic stress disorder section of the NIMH Diagnostic Interview Schedule Composite (DISC). Eighteen severely adverse events were chosen from these sources, modelled as closely as possible after Lovallo, et al. [66]. Both the DISC interview and the LEQ were completed by the participant and his/her parent. An event was considered an adverse life event if either the parent or participant confirmed that the event was experienced by the participant. For the LEQ, events were only considered an adverse life event if the informant coded the event as ‘unpleasant’ (for the participant). We excluded the entire scale from the child sample due to insufficient internal reliability.

#### Substance use-related factors

We assessed use of substances with the above-mentioned Health Questionnaire. *Caffeine use* was assessed with questions on ever use of cola and coffee. Similarly, ever use of *alcohol* (at least one glass) and *cannabis* were coded as yes (x = 1) or no (x = 0). *Tobacco use* was coded dichotomously as those who have never smoked, have smoked one or two cigarettes ever and currently smoke once in a while (x = 0); and those who smoke every day (x = 1). Those who had smoked in the past but had quit were excluded from analyses on tobacco use (N = 10). *Cola-use* was used the analyses in both child and adolescent samples, all other substance use items were used in the adolescent sample only.

### Statistical analysis

First, we computed descriptives for, as well as correlations between, all variables. All continuous predicting variables were centered. In order to confirm that the stressful tasks induced an increase in cortisol, heart rate and PPS, and a decrease in RSA as compared to the pre-task rest period, a manipulation check was performed by way of four repeated measures analyses of variances (RM-ANOVAs) in the child and adolescent samples separately. Departures from sphericity were corrected when necessary by reporting Greenhouse-Geisser statistics.

To systematically investigate determinants of the physiological and PPS reactivity measures, we first selected potential determinants based on earlier theories (i.e. [5], [6], [7]) and reviews (i.e. [8], [9], [10], [11]). Then, we conducted an exploratory, step-wise investigation (as in [84]) for each of the stress reactivity variables (cortisol, heart rate, RSA, PPS) in turn. In the first step of the analysis, we ran linear regressions with each of the potential determinants predicting each of the stress reactivity variables. If the results of the regression were significant at the *p*<.10 level, the potential determinant was entered into the subsequent multiple linear regression model (step 2). Before running the multiple linear regression model, we checked correlations between the significant potential determinants, and if any correlated strongly (r>.60), only one variable was included in the multiple linear regression model. In the second step, we confirmed which variables could be considered determinants of the stress reactivity variable. For this, we ran the multiple linear regression analysis with all potential determinants that were significant in the first step as predictors. Variables that were not significant (*p*>.10) were deleted from the model in a backwards step-wise manner, until only significant (*p*<.05) predictors were included. These steps were conducted for each of the stress reactivity variables.

Secondly, we examined the relation between physiological and PPS reactivity using three linear regressions in which the PPS response predicted each of the physiological stress responses. Variables were entered as covariates if they correlated significantly with both physiological and PPS reactivity variables. Covariates that were no longer significant in the model were dropped until the models contained only significant covariates. All analyses were performed in IBM SPSS statistics version 20.

## Results

Descriptives of all potential determinants are depicted in Table 1. Descriptives of all stress reactivity variables are depicted in Table 2, which showed large between-individual variation, allowing us to examine factors that contributed to this variation. In children, the parenting styles *involvement* and *positive parenting* were strongly correlated (r = .60), therefore only *positive parenting* was used in the analyses because it had stronger internal reliability. In adolescents, temperamental characteristics *shyness* and *sociabilit*y were strongly correlated (r = −.62), therefore only *sociability* was used in the analyses.

**Table 2:** Descriptives of stress reactivity variables.

	Children			Adolescents		
Variable	*N*	Range	*M*(*SD*)	*N*	Range	*M*(*SD*)
Cortisol reactivity	345	−5.20–19.00	1.77(3.27)	332	−15.98–13.94	0.65(3.31)
Heart rate reactivity	322	−6.39–34.94	8.58(7.01)	309	−10.69–39.95	10.21(8.44)
RSA reactivity	185	−1.96–3.40	0.34(0.69)	297	−2.06–1.92	0.05(0.62)
PPS reactivity	357	−11.00–42.00	4.87(6.90)	343	−8.00–34.00	6.77(6.76)

*Notes.* RSA = respiratory sinus arrhythmia; PPS = perceived physiological stress.

**Figure 2:**
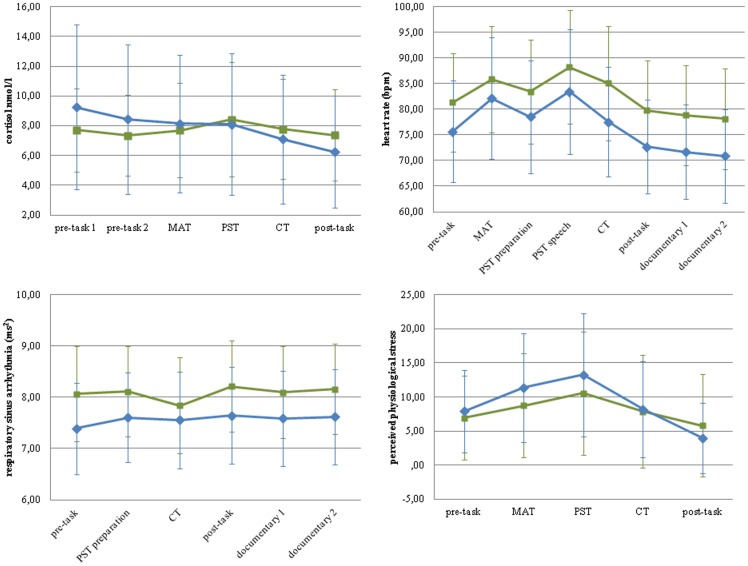
Raw data for each of the stress response variables across the psychosocial stress procedure in children and adolescents. *Notes.* MAT = mental arithmetic task; PST = public speaking task; CT = computer task; bpm = beats per minute.

In children, cortisol reactivity correlated significantly and positively with heart rate (r = .21, *p*<.001), but not RSA or PPS reactivity. Heart rate reactivity correlated significantly and positively with RSA (r = .35, *p*<.001) but not PPS reactivity. RSA and PPS reactivity were not significantly correlated. In adolescents, cortisol reactivity correlated significantly and positively with heart rate (r = .18, *p*<.01) and PPS (r = .13, *p*<.05), but not RSA reactivity. Heart rate reactivity correlated significantly and positively with RSA (r = .19, *p*<.01) and PPS (r = .11, *p*<.05) reactivity. RSA reactivity was not significantly correlated with PPS reactivity. Figure 2 illustrates the raw data for each of the stress reactivity variables across the psychosocial stress procedure, for each age group.

### Manipulation check

In the child sample, cortisol varied across periods, as evidenced by a significant within-subjects main effect of period, F(2.72,909.69) = 21.89, *p*<.001, η^2^ = .06. Simple contrasts showed significant increases in cortisol, relative to the pre-task rest, during the mental arithmetic task, F(1,334) = 5.87, *p*<.05, η^2^ = .02, the public speaking task, F(1,334) = 34.71, *p*<.001, η^2^ = .09 and the computer task F(1,334) = 4.98, *p*<.05, η^2^ = .02. Heart rate also varied across periods, as the within-subjects main effect of period was significant, F(3.95, 1232.25) = 303.70, *p*<.001, η^2^ = .49. Simple contrasts showed significant increases in heart rate, relative to the pre-task rest, during the mental arithmetic task, F(1,312) = 151.03, *p*<.001, η^2^ = .33, the public speaking task preparation, F(1,312) = 70.40, *p*<.001, η^2^ = .18, the public speaking task speech, F(1,312) = 287.94, p<.001, η^2^ = .48, and the computer task, F(1,312) = 121.49, p<.001, η^2^ = .28. RSA varied significantly across periods as well, showed by a significant within-subjects main effect of period F(4.15,701.82) = 16.12, *p*<.001, η^2^ = .09. Simple contrasts showed a significant decrease in RSA, relative to the pre-task rest, during the computer task, F(1,169) = 17.40, p<.001, η^2^ = .09, but not the public speaking preparation. Finally, the tasks were perceived as physiologically stressful, as evidenced by a significant within-subjects effect for PPS, F(3.18, 1111.31) = 66.14, *p*<.001, η^2^ = .16. Simple contrasts showed significant increases in PPS, relative to the pre-task rest, during the mental arithmetic task, F(1,349) = 47.61, *p*<.001, η^2^ = .12, the public speaking task F(1,349) = 98.31, p<.001, η^2^ = .22, and the computer task, F(1,349) = 9.92, p<.01, η^2^ = .03.

In the adolescent sample as a whole, cortisol varied across periods, as evidenced by a significant within-subjects main effect of period, F(2.37,763.26) = 83.01, *p*<.001, η^2^ = .21, however, not in the expected direction. Simple contrasts showed no significant difference between the pre-task rest period and the mental arithmetic task, and a significant decrease in cortisol during the public speaking task F(1,322) = 4.15, *p*<.05, η^2^ = .01 and the computer task F(1,322) = 57.33, *p*<.001, η^2^ = .15. This could be due to anticipation effects, which has previously been reported in cortisol studies (e.g. [54], [85]), so we examined the tasks relative to the post-task rest period. These contrasts showed that cortisol was increased, relative to the last period, during the mental arithmetic task, F(1,322) = 173.44, p<.001, η^2^ = .35, the public speaking task F(1,322) = 177.50, p<.001, η^2^ = .36, and the computer task, F(1,322) = 85.31, p<.001, η^2^ = .21. Heart rate also varied across periods, as the within-subjects main effect of period was significant, F(3.09, 922.02) = 326.31, *p*<.001, η^2^ = .52. Simple contrasts showed significant increases in heart rate, relative to the pre-task rest, during the mental arithmetic task, F(1,298) = 226.22, *p*<.001, η^2^ = .43, the public speaking task preparation, F(1,298) = 78.63, *p*<.001, η^2^ = .21, the public speaking task speech, F(1,298) = 242.48, p<.001, η^2^ = .45, and the computer task, F(1,298) = 31.47, p<.001, η^2^ = .10. RSA also varied across periods; the within-subjects main effect of period was significant; F(4.14,1075.75) = 11.97, p<.001, η^2^ = .04, though again, not in the expected direction. RSA increased (RSA augmentation) significantly during the tasks relative to the pre-task rest (public speaking task preparation: F(1,260) = 37.12, p<.001, η^2^ = .13; computer task: F(1,260) = 13.23, p<.001, η^2^ = .05) as opposed to the expected RSA withdrawal. This was also likely due to anticipation effects (see also [14]), so we examined the tasks relative to the post-task rest period. These contrasts showed that RSA was not significantly decreased during the public speaking preparation and computer tasks, relative to the sixth period. The tasks were perceived as physiologically stressful, as evidenced by a significant within-subjects effect for PPS, F(3.07, 1040.22) = 238.10, *p*<.001, η^2^ = .41. Simple contrasts showed significant increases in PPS, relative to the pre-task rest, during the mental arithmetic task, F(1,339) = 125.14, *p*<.001, η^2^ = .27, and the public speaking task F(1,339) = 195.72, p<.001, η^2^ = .37, but not the computer task.

### Determinants of the stress response

An overview of the results of the linear regressions for each potential determinant predicting each of the stress responses for both children and adolescents is portrayed in Table 3. Tables 4 and 5 depict the results of the four multiple linear regression models for children and adolescents, respectively.

**Table 3:** Results of the linear regression models predicting each stress response in children and adolescents.

Sample	Children				Adolescents			
Reactivity variable	Cortisol	Heart rate	RSA	PPS	Cortisol	Heart rate	RSA	PPS
**Predictor**	β(*p*)	β(*p*)	β(*p*)	β(*p*)	β(*p*)	β(*p*)	β(*p*)	β(*p*)
**Individual:** Sex	**.17(<.01)**	**.10(<.10)**	−.07(.36)	−.01(.87)	**−.12(<.05)**	**.10(<.10)**	.05(.38)	.06(.27)
Ethnicity	−.03(.62)	−.08(.14)	.01(.85)	−.02(.76)	−.06(.32)	**−.13(<.05)**	.01(.87)	−.08(.15)
Body mass index	−.07(.17)	**−.15(<.05)**	**−.17(<.05)**	.05(.31)	−.01(.80)	**−.12(<.05)**	.02(.76)	−.05(.36)
Birth weight	−.09(.12)	.04(.54)	−.09(.25)	−.08(.15)	.10(.14)	−.01(.84)	−.06(.34)	.04(.52)
Menstrual cycle phase					.03(.83)	−.03(.80)	.07(.62)	.03(.79)
Oral contraceptive use					**−.14(<.10)**	**−.14(<.10)**	**−.20(<.05)**	−.01(.91)
Shyness	.04(.54)	.03(.64)	−.03(.72)	**.16(<.05)**				
Activity	.03(.61)	−.02(.80)	.08(.34)	**−.13(<.05)**	−.07(.23)	−.08(.20)	**−.14(<.05)**	.02(.72)
Emotionality	−.04(.52)	−.04(.61)	.10(.22)	.06(.32)	**−.13(<.05)**	−.04(.49)	−.02(.75)	.03(.56)
Sociability	−.04(.49)	.09(.18)	.09(.30)	**−.12(<.05)**	**−.14(<.05)**	**−.16(<.01)**	**−.11(<.10)**	.04(.51)
**Development:** Age	**.09(<.10)**	−.04(.45)	**−.17(<.05)**	**.15(<.01)**	**−.12(<.05)**	−.03(.59)	−.02(.78)	−.05(.34)
Pubertal stage					−.09(.14)	.02(.79)	.00(.94)	−.07(.24)
Menstruation	−.03(.74)	**−.17(<.05)**	.01(.93)	−.04(.58)				
**Environ:** Urbanicity	.01(.80)	**−.15(<.01)**	.07(.35)	.00(.96)	**−.13(<.05)**	**−.22(<.001)**	−.07(.23)	.02(.72)
Socioeconomic status	.03(.60)	**.15(<.01)**	**.17(<.05)**	.05(.36)	−.07(.23)	**.23(<.001)**	.05(.41)	.01(.83)
Family situation	.05(.46)	.03(.72)	−.07(.41)	**.11(<.10)**	.07(.25)	**−.15(<.05)**	.05(.42)	−.01(.84)
Emotional warmth	**.17(<.01)**	.09(.13)	−.07(.33)	−.02(.73)	**.12(<.05)**	**.11(<.10)**	−.04(.54)	.04(.48)
Overprotection	−.06(.27)	−.03(.64)	−.03(.66)	.07(.23)	−.02(.74)	.04(.52)	.08(.20)	**.14(<.05)**
Rejection	**−.12(<.05)**	.05(.42)	.11(.16)	**.09(<.10)**	−.02(.77)	−.01(.88)	.05(.39)	−.01(.85)
Involvement					**.14(<.05)**	**.16(<.01)**	.06(.36)	−.03(.63)
Positive parenting	−.02(.78)	**.12(<.10)**	.05(.56)	−.06(.34)	−.01(.88)	.01(.94)	−.07(.26)	−.04(.49)
Inconsistent discipline	−.08(.23)	.03(.61)	.01(.92)	−.03(.64)	**−.10(<.10)**	−.04(.50)	.00(.97)	**−.10(<.10)**
Corporal punishment	−.03(.68)	−.04(.59)	.03(.71)	−.10(.10)				
Adverse life events					.05(.37)	−.08(.18)	−.07(.24)	.06(.30)
**Substance use:** Cola	−.04(.49)	**−.10(<.10)**	−.05(.54)	−.02(.75)	−.01(.88)	−.01(.90)	**−.11(<.10)**	−.02(.66)
Coffee use					−.06(.28)	.01(.91)	.08(.15)	−.03(.60)
Tobacco use					**−.17(<.01)**	**−.22(<.001)**	−.09(.12)	**−.11(<.05)**
Alcohol use					−.03(.64)	.09(.14)	−.03(.67)	.07(.22)
Cannabis use					−.04(.45)	**−.10(<.10)**	−.04(.47)	.02(.74)

*Notes.* RSA = respiratory sinus arrhythmia; PPS = perceived physiological stress; β refers to standardized coefficients; **bold** = *p*<.10.

**Table 4:** Results of the multiple linear regression models, predicting each stress response in the child sample.

	F (*p*) or β(*p*)	R^2^
**Cortisol reactivity**	8.32 (<.001)	.04
Sex	.14 (<.05)	
Emotional warmth	.14 (<.05)	
**Heart rate reactivity**	8.19 (<.001)	.04
Urbanicity	−.17 (<.01)	
SES	.16 (<.01)	
**RSA reactivity**	5.34(<.01)	.05
Age	−.16(<.05)	
SES	.17(<.05)	
**PPS reactivity**	6.04(<.01)	.06
Shyness	.17(<.01)	
Age	.14(<.05)	
Rejection	.15(<.05)	

*Notes.* SES = socioeconomic status; RSA = respiratory sinus arrhythmia; PPS = perceived physiological stress; adjusted R^2^ reported; sample sizes for each reactivity model: cortisol (*n* = 336), heart rate (*n* = 320); RSA (*n* = 184); PPS (*n* = 251); F statistics pertain to model results, β statistics refer to standardized coefficients of individual predictors.

**Table 5:** Results of the multiple linear regression models, predicting each stress response in the adolescent sample.

	F(*p*) or β(*p*)	R^2^
**Cortisol reactivity**	6.07(<.001)	.06
Sociability	−.15(<.01)	
Emotionality	−.14(<.05)	
Urbanicity	−.12(<.05)	
Involvement	.16(<.01)	
**Heart rate reactivity**	9.39(<.001)	.16
Sex	.13(<.05)	
Sociability	−.14(<.05)	
Urbanicity	−.20(<.001)	
SES	.18(<.01)	
Involvement	.12(<.05)	
Tobacco use	−.17(<.01)	
**RSA reactivity**	5.71(<.05)	.02
Activity	−.14(<.05)	
**PPS reactivity**	6.10(<.001)	.05
Overprotection	.19(<.01)	
Inconsistent discipline	−.14(<.05)	
Tobacco use	−.13(<.05)	

*Notes.* SES = socioeconomic status; RSA = respiratory sinus arrhythmia; PPS = perceived physiological stress; adjusted R^2^ reported; sample sizes for each reactivity model: cortisol (*n* = 302), heart rate (*n* = 265); RSA (*n* = 270); PPS (*n* = 287); F statistics pertain to model results, β statistics refer to standardized coefficients of individual predictors.

#### Cortisol reactivity

In children, *sex*, *age*, and parenting styles *emotional warmth* and *rejection* significantly predicted cortisol reactivity in the exploratory linear regression analyses. In the confirmatory, final model, individuals with male *sex*, who perceived less *emotional warmth* from their parents portrayed lower cortisol reactivity to stress. In adolescents, *sex*, *oral contraceptive use*, *emotionality*, *sociability*, *age*, *urbanicity*, parenting styles *emotional warmth*, *involvement* and *inconsistent discipline*, and *tobacco use* significantly predicted cortisol reactivity independently. In the final model, individuals who scored higher in *emotionality* and *sociability*, who lived in more *urban* areas, and whose parents showed less *involvement* portrayed blunted cortisol reactivity. Because *oral contraceptive use* was a significant factor in the first step, we reran the final model in girls only, and *oral contraceptive use* did not remain a significant determinant. In sum, cortisol reactivity was determined by *sex* and perceived parental *emotional warmth* in children, and *emotionality*, *sociability*, *urbanicity*, and parental *involvement* in adolescents.

#### Heart rate reactivity

In children, *sex*, *BMI*, *menstruation*, *urbanicity*, *SES*, parenting style *positive parenting*, and *cola use* were the initial significant predictors of heart rate reactivity. The final model showed that having a lower *SES* and a higher *urbanicity* score predicted lower heart rate reactivity to stress. In the final model in girls only, *menstruation* did not remain a significant predictor. In adolescents, *sex*, *ethnicity*, *BMI*, *oral contraceptive use*, *sociability*, *urbanicity*, *SES*, *family situation*, parenting styles *emotional warmth* and *involvement*, and *tobacco* and *cannabis use* were initially significant predictors of heart rate reactivity. In the final model, individuals with male *sex*, who were more *sociable*, lived in more *urban* areas, had a lower *SES*, whose parents were less *involved*, and who were more likely to *use tobacco* daily portrayed blunted heart rate reactivity. In girls only, *oral contraceptive use* did not remain a significant predictor. In sum, heart rate reactivity was determined by *urbanicity* and *SES* in children, and *sex*, *sociability*, *urbanicity*, *SES*, parental *involvement* and *tobacco use* in adolescents.

#### Respiratory sinus arrhythmia reactivity

In children, *BMI*, *age*, and *SES* significantly predicted RSA reactivity in the exploratory phase. In the final model, older *age* and lower *SES* were related to less pronounced RSA reactivity (i.e. RSA decreased less in response to the tasks). In adolescents, *oral contraceptive use*, *activity*, *sociability*, and *cola use* significantly predicted RSA reactivity. In the final model, individuals who were more *active* portrayed less pronounced RSA reactivity. *Oral contraceptive* use did not remain significant in the final model in girls only. In sum, RSA reactivity was determined by *age* and *SES* in children, and *activity* level in adolescents.

#### Perceived physiological stress reactivity

In children, *shyness*, *activity*, *sociability*, *age*, *family situation*, and parental *rejection* significantly predicted PPS reactivity initially. In the final model, a lower level of *shyness*, younger *age* and lower perceived parental *rejection* were related to lower PPS reactivity. In adolescents, parenting styles *overprotection* and *inconsistent discipline* and *tobacco use* were significantly related to PPS reactivity in the exploratory phase. In the final model, lower perceived parental *overprotection*, higher parent-reported *inconsistent discipline*, and a higher likelihood of daily *tobacco use* were related to lower PPS reactivity. In sum, PPS reactivity was determined by *shyness*, *age* and perceived parental *rejection* in children, and perceived parental *overprotection*, parental *inconsistent discipline* and *tobacco use* in adolescents.

### Perceived physiological and physiological stress reactivity

In children, PPS reactivity was not significantly related to cortisol, heart rate or RSA reactivity, although all of these relations were marginally significant and positive (all *p*s<.13). In adolescents, PPS reactivity significantly predicted cortisol reactivity (*b* = .13, *p*<.05, controlling for tobacco use), but not heart rate or RSA reactivity, though these relations were also marginally significant and positive (*p*s<.10).

## Discussion

**Figure 3:**
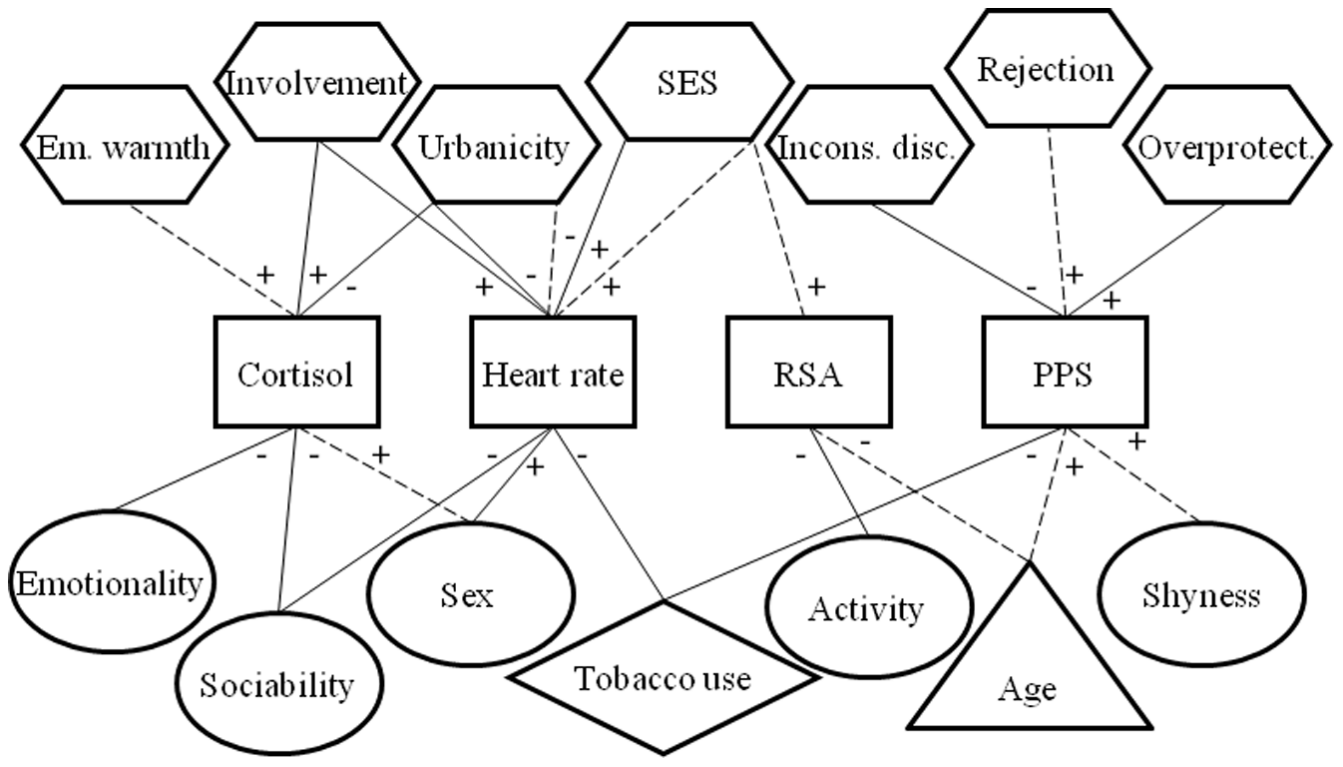
Schematic description of significant determinants of the four indices of the stress response. *Notes.* Em. = emotional; SES = socioeconomic status; Incons. disc. = inconsistent discipline; RSA = respiratory sinus arrhythmia; PPS = perceived physiological stress; square outlines pertain to stress responses; circles pertain to individual factors; triangles pertain to developmental factors; hexagons pertain to environmental factors; diamonds pertain to substance use- related factors; dotted connecting lines pertain to children; solid connecting lines pertain to adolescents.

This study investigated physiological and PPS reactivity to psychosocial stress in 363 children and 344 adolescents from the general population. The first aim of this study was to systematically examine potential determinants of stress reactivity (i.e. cortisol, heart rate, RSA and PPS). Multivariate regression models showed distinct determinants for each of the stress response indices, and for children versus adolescents. Cortisol reactivity was related to *sex* and perceived parental *emotional warmth* in children, and *emotionality*, *sociability*, *urbanicity* and parental *involvement* in adolescents. Heart rate reactivity was related to *urbanicity* and *SES* in both children and adolescents, and furthermore *sex*, *sociability*, parental *involvement* and *tobacco use* in adolescents. RSA reactivity was related to *age* and *SES* in children, and *activity* level in adolescents. PPS reactivity was related to *shyness*, *age* and perceived parental *rejection* in children, and perceived parental *overprotection*, parent-reported *inconsistent discipline* and *tobacco use* in adolescents. Please see Figure 3 for a graphic representation of these results.

The second part of this study examined the associations between physiological and PPS reactivity. We observed that PPS reactivity significantly predicted cortisol reactivity in adolescents only, but none of the other physiological stress responses in children or adolescents, although these relations were marginally significant (all *p*s were between .05 and .12). This is only minimally in accordance with a recent study that confirmed the covariation between PPS and cortisol, RSA and heart rate reactivity in a large sample of adolescents from the general population, though effect sizes were small [14]. In our study, though, when children and adolescents were pooled, PPS did predict each of the physiological stress response indices, albeit not strongly (data not shown). We therefore conclude that physiological and PPS reactivity may be marginally positively related, such that these relations are only detectable in large samples (as the Oldehinkel study and our own with children and adolescents pooled are). When we examined children and adolescents separately, we likely had insufficient power to detect such weak relations.

Intriguingly, we observed differential results for the manipulation check in children versus adolescents. In children, the physiological response patterns were as we would expect during a psychosocial stress procedure, that is, relative to the pre-task rest period, cortisol, heart rate and PPS increased, and RSA decreased during the stressful tasks. In adolescents, however, only heart rate and PPS showed this expected pattern. Cortisol and RSA levels were highest/lowest during the pre-task rest period, and did not increase/decrease further in response to the tasks. Physiological anticipation has been frequently reported in the literature (e.g. [14], [54], [85]), and therefore was not entirely surprising. Moreover, RSA withdrawal entails the most immediate response to an anticipated stressor [21], and the cortisol response is also strongly associated with anticipatory stress appraisal [86]. Most interesting was that this anticipation effect was observed only in adolescents. Executive functions (i.e. planning, cognitive flexibility, working memory) develop increasingly during adolescence [87], and could explain our findings. Perhaps adolescents anticipate the upcoming stressor more than children do, and begin to plan subsequent reactions or behavior during the pre-task rest, thus eliciting a strong physiological anticipatory response. Adolescents did not report *perceiving* more physiological stress prior to the tasks (PPS response patterns were similar in children and adolescents), which indicates that this anticipation effect was physiological; it comprised both the autonomic nervous system (though only RSA) and HPA systems. Animal studies showed dramatic differences in the physiological stress systems in adolescents as compared to adults [16], and more research on the developmental of these systems in humans is imperative to our understanding of these systems as vulnerability markers for (mental) health problems.

### Determinants of the stress response

#### Individual factors

In our study we found that *sex* was related to stress reactivity, with girls showing higher heart rate and cortisol reactivity, which is consistent with earlier findings pertaining to heart rate, however sex differences in cortisol reactivity are generally not found in children [31], [32]. *Oral contraceptive use* and *menstrual cycle phase* were unrelated to stress reactivity. These factors were shown to be of influence in adults (i.e. [35], [37]), but not in adolescents [34], perhaps because the menstrual cycle of girls is less stable, or a different ratio of sex hormones contribute differently to cortisol reactivity [34]. PPS reactivity was found in our study not to vary according to sex which is consistent with previous studies [33], [34].

In line with theoretical propositions [46], [47], [48], we found effects of *activity* level, *emotionality*, *shyness* and *sociability* on cortisol, heart rate, RSA and PPS reactivity. Specifically, in children, decreased *shyness* was related to lower PPS reactivity. In adolescents, a higher *sociability* score was related to lower cortisol and heart rate reactivity. A higher *emotionality* score was also related to lower cortisol reactivity, and a higher *activity* level was related to lower RSA reactivity. Previous research would suggest a strong relation between temperament measures and physiological stress reactivity [88], which we found, interestingly, for adolescents but not children, as only *shyness* was related to PPS reactivity in children. Possibly, in children, the effects of temperament on stress responses were outweighed by other determinants included in the models. This underlines the importance of examining multiple determinants of physiological reactivity in a single study. Previous studies in young children and adults have also reported significant effects of temperament on physiological stress reactivity [89], [90]. It is unclear why these relations should be of greater importance in adolescence as opposed to late childhood, and this warrants further research. The other individual factors that were examined in this study (i.e. *ethnicity*, *BMI* and *birth weight*) were not related to any of the physiological or PPS responses.

#### Developmental factors

Because of the large sample in the present study, we were able to examine determinants of stress reactivity in children and adolescents separately. The physiological stress systems are proposed to undergo developmental changes between childhood and adolescence [16], and indeed, our data showed distinct determinants influencing stress reactivity in childhood versus adolescence. Only the influence of urbanicity and SES on heart rate reactivity was uniform in both samples. In general, pertaining to physiological stress alone, the physiological reactivity indices (heart rate with cortisol and RSA) were less strongly correlated in adolescents as compared to children. Also, the manipulation check showed that the stress response patterns were as expected in children, but not in adolescents. Pertaining to the determinants, we observed that temperament seemed to be a more important influence in adolescents than in children. Age remained a significant factor in children, but not in adolescents. We were particularly interested in pubertal development, as some previous studies suggested this might be important (e.g. [33]), but this did not remain a significant factor in adolescents our study. In children, we examined *menstruation* in girls as a proxy for pubertal development, but this was also not related to stress reactivity. We observed significant effects of parenting in both children and adolescents, albeit different parenting styles. In sum, the differential response patterns and differential determinants of stress reactivity in children and adolescents found in our study confirm previous propositions of developmental changes in physiological stress reactivity in humans during adolescence [16], [51].

#### Environmental factors

Environmental determinants of the stress response have been neither frequently examined nor controlled for in past studies. This study showed that such factors may, however, be crucial variables, accounting for significant variability in all stress responses. Initial hypotheses in the literature proposed that (early life) adversities are associated with physiological hyper-reactivity to stress (e.g. [55]). However, there also seems to be evidence in animal and human research that early environmental adversity is linked to subsequent blunted physiological stress responses (i.e. [91], [92]). The findings from this study generally support this latter hypothesis, as we found that a lower *SES* and living in a more *urban* area were related to blunted heart rate reactivity in children and adolescents, blunted RSA reactivity in children and blunted cortisol reactivity in adolescents, confirming earlier findings [40]. As lower *SES* environments and city-dwelling are viewed as a more demanding and socially stressful, these could be considered more adverse environments to grow up in, thus blunting stress responses. Alternatively, given that physiological stress systems are partially genetically determined, it is possible that (the parents of) individuals who are inherently hypo-aroused, move to, for example, more populated areas or attain a lower *SES*.

Parenting styles index the family environment of youth and in this way may influence the developing stress systems. Protective parenting factors were related to increased cortisol, heart rate and PPS reactivity in the present study. This is consistent with earlier findings of less optimal parenting behaviors being related to HPA hypo-reactivity [62]. In our study, less *emotional warmth* was related to blunted cortisol reactivity in children, less *involvement* was related to blunted cortisol and heart rate reactivity, and less *overprotection* was related to blunted PPS reactivity in adolescents. Comparably, a higher degree of *inconsistent discipline* was related to blunted PPS reactivity in adolescents. The less optimal perceived parental *rejection* was positively related to PPS reactivity in children, which does not fit with this pattern. However, this pertains to *perceived* parental rejection, and likewise, *perceived* physiological stress. That this relation is positive, may indicate that some children are more sensitive to external influences, and for this reason reported a higher degree of both parental *rejection* and PPS during the psychosocial stress procedure.

(Early) adversity may also be indexed by number of *adverse life events*. Previous studies found that having experienced more adverse life events was related to blunted cortisol and heart rate reactivity in boys [42], [64] and young adults [65], [66]. In contrast to these findings, we observed no relation between adverse life events and physiological stress responses in adolescents. Internal reliability was, however, quite low in the adolescent sample, which could account for these findings.

#### Substance use-related factors

In our study, in the adolescent sample, we found *tobacco use* to be associated with blunted cortisol and PPS reactivity. The former is consistent with previous research [67], [93]. Previous studies also found *alcohol* and *cannabis use* to be related to blunted cortisol and heart rate reactivity [67], [94], though these relations were not evident in the present study. An effect of *tobacco use* on physiological reactivity was expected, yet the finding of its effect on PPS seems less intuitive, and has not been previously examined to our knowledge. It is not entirely clear why we observed this relation in the present study, and further research will be needed to confirm or disprove this finding.

The results of this study should be considered in light of some limitations. First, the study was cross-sectional, therefore no conclusions can be drawn as to the direction of influence of the determinants and the stress reactivity variables. Second, information on pubertal stage was only available for the adolescent sample. We were able to assess puberty-related changes in girls in the child sample with *menstruation*, however, it would have been preferable to have information on pubertal stage in children as well. Developmental differences within the child and adolescent samples are likely to have still been influential, as the age ranges within these samples were still quite large. In order to fully examine developmental differences in relation to stress reactivity, it is necessary to have a sufficiently large sample to divide it into groups of smaller age ranges. Third, *SES* was operationalized based on the higher occupational level of either parent and coded into only three levels. Furthermore, only a small percentage of participants had a low *SES* background. A more comprehensive definition of *SES*, including financial, educational as well as occupational information is necessary to fully examine the relation of *SES* with stress response indices. Fourth, though our PPS questionnaire has been used in previous studies, it has not yet been formally validated. Fifth, our measures of stress reactivity were based on difference scores. Such measures are still widely used and clearly superior to single measurements, however, more comprehensive measures that utilize all of the measurement points, such as area under the curve estimates or growth curve modeling, are more sophisticated than difference scores [95] and preferable in stress reactivity research.

Current research has focused increasingly on physiological stress reactivity as a vulnerability marker for (mental) health problems in children and adolescents. However, such studies are inconsistent in the inclusion of potential covariates that may influence the developing stress systems. Determinant factors of stress reactivity were outlined in several theories (e.g. [5], [6], [7]), and although several reviews of such determinants are available (i.e. [8], [9], [10], [11]), our sample of children and adolescents from the general population provided an exceptional opportunity to systematically test such factors, also as reports in the literature pertaining to many of these factors are either lacking or inconclusive. In children and adolescents, individual, developmental, environmental and substance use-related factors influenced stress reactivity measures. Furthermore, we investigated the hypothesis that PPS reactivity was positively related to cortisol, heart rate and RSA reactivity, but found that, for the most part, this did not hold in our samples, in contrast to a recent study in adolescents [14]. In sum, our study showed that it is imperative that future studies take into consideration determinants of stress reactivity that may account for found relations. This study provides an indication of which determinants should be considered in children and adolescents.

## References

[1] Lovallo WR (2011) Do low levels of stress reactivity signal poor states of health? Biological Psychology 86: 121–128. 20079397 10.1016/j.biopsycho.2010.01.006 PMC2888887

[2] Greaves-Lord K, Ferdinand RF, Sondeijker FEPL, Dietrich A, Oldehinkel AJ, et al (2007) Testing the tripartite model in young adolescents: Is hyperarousal specific for anxiety and not depression? Journal of Affective Disorders 102: 55–63. 17234274 10.1016/j.jad.2006.12.009

[3] Kagan J, Reznick JS, Snidman N (1987) The physiology and psychology of behavioral-inhibition in children. Child Development 58: 1459–1473. 3691195

[4] Ortiz J, Raine A (2004) Heart rate level and antisocial behavior in children and adolescents: A meta-analysis. Journal of the American Academy of Child & Adolescent Psychiatry 43: 154–162. 14726721 10.1097/00004583-200402000-00010

[5] McEwen BS (1998) Protective and damaging effects of stress mediators. New England Journal of Medicine 338: 171–179. 9428819 10.1056/NEJM199801153380307

[6] Alpert BS, Wilson DK (1992) Stress reactivity in childhood and adolescence. In: Turner JR, Sherwood A, Light KC, editors. Individual differences in cardiovascular response to stress. New York, NY: Plenum Press. pp. 187–198.

[7] Lazarus R, Folkman S (1984) Stress, Appraisal, and Coping. New York, NY: Springer Publishing Company, Inc.

[8] Kirschbaum C, Hellhammer DH (1989) Salivary cortisol in psychobiological research - an overview. Neuropsychobiology 22: 150–169. 2485862 10.1159/000118611

[9] Jessop DS, Turner-Cobb JM (2008) Measurement and meaning of salivary cortisol: A focus on health and disease in children. Stress: The International Journal on the Biology of Stress 11: 1–14. 10.1080/10253890701365527 17853059

[10] Kudielka BM, Wüst S (2010) Human models in acute and chronic stress: Assessing determinants of individual hypothalamus–pituitary–adrenal axis activity and reactivity. Stress: The International Journal on the Biology of Stress 13: 1–14. 10.3109/10253890902874913 20105052

[11] Kudielka BM, Hellhammer DH, Wüst S (2009) Why do we respond so differently? Reviewing determinants of human salivary cortisol responses to challenge. Psychoneuroendocrinology 34: 2–18. 19041187 10.1016/j.psyneuen.2008.10.004

[12] Schommer NC, Hellhammer DH, Kirschbaum C (2003) Dissociation between reactivity of the hypothalamus-pituitary-adrenal axis and the sympathetic-adrenal-medullary system to repeated psychosocial stress. Psychosomatic Medicine 65: 450–460. 12764219 10.1097/01.psy.0000035721.12441.17

[13] Thayer RE (1970) Activation states as assessed by verbal report and four psychophysiological variables. Psychophysiology 7: 86–94. 5492738 10.1111/j.1469-8986.1970.tb02278.x

[14] Oldehinkel AJ, Ormel J, Bosch NM, Bouma EMC, Van Roon AM, et al (2011) Stressed out? Associations between perceived and physiological stress responses in adolescents: The TRAILS study. Psychophysiology 48: 441–452. 21361964 10.1111/j.1469-8986.2010.01118.x

[15] Bauer AM, Quas JA, Boyce WT (2002) Associations between physiological reactivity and children's behavior: Advantages of a multisystem approach. Journal of Developmental and Behavioral Pediatrics 23: 102–113. 11943973 10.1097/00004703-200204000-00007

[16] Romeo RD (2010) Adolescence: A Central Event in Shaping Stress Reactivity. Developmental Psychobiology 52: 244–253. 20175102 10.1002/dev.20437

[17] Spear LP (2009) Heightened stress responsivity and emotional reactivity during pubertal maturation: Implications for psychopathology. Development and Psychopathology 21: 87–97. 19144224 10.1017/S0954579409000066 PMC3036838

[18] Hollenstein T, McNeely A, Eastabrook J, Mackey A, Flynn J (2012) Sympathetic and parasympathetic responses to social stress across adolescence. Developmental Psychobiology 54: 207–214. 21688260 10.1002/dev.20582

[19] Rosmalen JGM, Oldehinkel AJ, Ormel J, de Winter AF, Buitelaar JK, et al (2005) Determinants of salivary cortisol levels in 10–12 year old children; a population-based study of individual differences. Psychoneuroendocrinology 30: 483–495. 15721059 10.1016/j.psyneuen.2004.12.007

[20] Porges SW (1995) Cardiac vagal tone: A physiological index of stress. Neuroscience & Biobehavioral Reviews 19: 225–233. 7630578 10.1016/0149-7634(94)00066-a

[21] Porges SW (2007) The polyvagal perspective. Biological Psychology 74: 116–143. 17049418 10.1016/j.biopsycho.2006.06.009 PMC1868418

[22] Tornhage CJ (2009) Salivary Cortisol for Assessment of Hypothalamic-Pituitary-Adrenal Axis Function. Neuroimmunomodulation 16: 284–289. 19571589 10.1159/000216186

[23] Seyle H (1950) Stress: The physiology and pathology of exposure to stress. Montreal: Acta.

[24] Klimes-Dougan B, Hastings PD, Granger DA, Usher BA, Zahn-Waxler C (2001) Adrenocortical activity in at-risk and normally developing adolescents: Individual differences in salivary cortisol basal levels, diurnal variation, and responses to social challenges. Development and Psychopathology 13: 695–719. 11523855 10.1017/s0954579401003157

[25] Munck A, Guyre PM, Holbrook NJ (1984) Physiological functions of glucocorticoids in stress and their relation to pharmacological actions. Endocrine Reviews 5: 25–44. 6368214 10.1210/edrv-5-1-25

[26] Lovallo WR (2005) Stress and health: Biological and psychological interactions. Thousand Oaks, CA: Sage Publications, Inc.

[27] Kirschbaum C, Pirke K-M, Hellhammer DH (1993) The ‘Trier Social Stress Test’: A tool for investigating psychobiological stress responses in a laboratory setting. Neuropsychobiology 28: 76–81. 8255414 10.1159/000119004

[28] Kirschbaum C (2010) Trier social stress test. In: Stolerman IP, editor. Encyclopedia of psychopharmacology: Trier social stress test. Berlin Heidelberg: Springer-Verlag.

[29] Dickerson SS, Kemeny ME (2004) Acute stressors and cortisol responses: A theoretical integration and synthesis of laboratory research. Psychological Bulletin 130: 355–391. 15122924 10.1037/0033-2909.130.3.355

[30] Kajantie E, Phillips DIW (2006) The effects of sex and hormonal status on the physiological response to acute psychosocial stress. Psychoneuroendocrinology 31: 151–178. 16139959 10.1016/j.psyneuen.2005.07.002

[31] Kudielka BM, Buske-Kirschbaum A, Hellhammer DH, Kirschbaum C (2004) HPA axis responses to laboratory psychosocial stress in healthy elderly adults, younger adults, and children: Impact of age and gender. Psychoneuroendocrinology 29: 83–98. 14575731 10.1016/s0306-4530(02)00146-4

[32] Dockray S, Susman EJ, Dorn LD (2009) Depression, cortisol reactivity, and obesity in childhood and adolescence. Journal of Adolescent Health 45: 344–350. 19766938 10.1016/j.jadohealth.2009.06.014 PMC2750854

[33] Gunnar MR, Wewerka S, Frenn K, Long JD, Griggs C (2009) Developmental changes in hypothalamus-pituitary-adrenal activity over the transition to adolescence: Normative changes and associations with puberty. Development and Psychopathology 21: 69–85. 19144223 10.1017/S0954579409000054 PMC3933029

[34] Bouma EMC, Riese H, Ormel J, Verhulst FC, Oldehinkel AJ (2009) Adolescents' cortisol responses to awakening and social stress; Effects of gender, menstrual phase and oral contraceptives. The TRAILS study. Psychoneuroendocrinology 34: 884–893. 19195792 10.1016/j.psyneuen.2009.01.003

[35] Kirschbaum CP, Kudielka BMMS, Gaab JMS, Schommer NCMS, Hellhammer DHP (1999) Impact of gender, menstrual cycle phase, and oral contraceptives on the activity of the hypothalamus-pituitary-adrenal axis. SO - Psychosomatic Medicine March/April 1999 61 (2) 154–162. 10.1097/00006842-199903000-00006 10204967

[36] Childs E, Dlugos A, De Wit H (2010) Cardiovascular, hormonal, and emotional responses to the TSST in relation to sex and menstrual cycle phase. Psychophysiology 47: 550–559. 20070572 10.1111/j.1469-8986.2009.00961.x PMC4242596

[37] Rohleder N, Wolf JM, Piel M, Kirschbaum C (2003) Impact of oral contraceptive use on glucocorticoid sensitivity of pro-inflammatory cytokine production after psychosocial stress. Psychoneuroendocrinology 28: 261–273. 12573295 10.1016/s0306-4530(02)00019-7

[38] Girdler SS, Jamner LD, Jarvik M, Soles JR, Shapiro D (1997) Smoking status and nicotine administration differentially modify hemodynamic stress reactivity in men and women. Psychosomatic Medicine 59: 294–306. 9178340 10.1097/00006842-199705000-00012

[39] Straneva P, Hinderliter A, Wells E, Lenahan H, Girdler S (2000) Smoking, oral contraceptives, and cardiovascular reactivity to stress. Obstetrics and Gynecology 95: 78–83. 10636507 10.1016/s0029-7844(99)00497-4

[40] Musante L, Treiber FA, Kapuku G, Moore D, Davis H, et al (2000) The effects of life events on cardiovascular reactivity to behavioral stressors as a function of socioeconomic status, ethnicity, and sex. Psychosomatic Medicine 62: 760–767. 11138994 10.1097/00006842-200011000-00004

[41] Roemmich JN, Smith JR, Epstein LH, Lambiase M (2007) Stress reactivity and adiposity of youth. Obesity 15: 2303–2310. 17890499 10.1038/oby.2007.273

[42] Boyce WT, Chesterman E (1990) Life events, social support, and cardiovascular reactivity in adolescence. Journal of Developmental and Behavioral Pediatrics 11: 105–111. 2365830

[43] Wust S, Entringer S, Federenko IS, Schlotz W, Hellhammer DH (2005) Birth weight is associated with salivary cortisol responses to psychosocial. stress in adult life. Psychoneuroendocrinology 30: 591–598. 15808929 10.1016/j.psyneuen.2005.01.008

[44] Kajantie E, Raikkonen K (2010) Early life predictors of the physiological stress response later in life. Neuroscience and Biobehavioral Reviews 35: 23–32. 19931557 10.1016/j.neubiorev.2009.11.013

[45] Jones A, Godfrey KM, Wood P, Osmond C, Goulden P, et al (2006) Fetal growth and the adrenocortical response to psychological stress. Journal of Clinical Endocrinology & Metabolism 91: 1868–1871. 16464950 10.1210/jc.2005-2077

[46] Goldsmith HH, Buss AH, Plomin R, Rothbart MK, Thomas A, et al (1987) Round table: What is temperament: 4 approaches. Child Development 58: 505–529. 3829791

[47] Derryberry D, Rothbart MK (1988) Arousal, affect, and attention as components of temperament. Journal of Personality and Social Psychology 55: 958–966. 3216290 10.1037//0022-3514.55.6.958

[48] Kagan J (1997) Temperament and the reactions to unfamiliarity. Child Development 68: 139–143. 9084130

[49] Doussard-Roosevelt JA, Montgomery LA, Porges SW (2003) Short-term stability of physiological measures in kindergarten children: Respiratory sinus arrhythmia, heart period, and cortisol. Developmental Psychobiology 43: 230–242. 14558045 10.1002/dev.10136

[50] Boyce WT, Barr RG, Zeltzer LK (1992) Temperament and the psychobiology of childhood stress. Pediatrics 90: 483–486. 1513612

[51] Del Giudice M, Ellis BJ, Shirtcliff EA (2011) The Adaptive Calibration Model of stress responsivity. Neuroscience and Biobehavioral Reviews 35: 1562–1592. 21145350 10.1016/j.neubiorev.2010.11.007 PMC3068241

[52] Stroud LR, Foster E, Papandonatos GD, Handwerger K, Granger DA, et al (2009) Stress response and the adolescent transition: Performance versus peer rejection stressors. Development and Psychopathology 21: 47–68. 19144222 10.1017/S0954579409000042 PMC2700625

[53] Susman E, Rogol A (2004) Puberty and psychological development. In: Lerner RM, Steinberg L, editors. Handbook of adolescent psychology. Second ed. Hoboken, New Jersey: John Wiley & Sons, Inc. pp. 15–44.

[54] Sumter SR, Bokhorst CL, Miers AC, Van Pelt J, Westenberg PM (2010) Age and puberty differences in stress responses during a public speaking task: Do adolescents grow more sensitive to social evaluation? Psychoneuroendocrinology 35: 1510–1516. 20541871 10.1016/j.psyneuen.2010.05.004

[55] Gunnar MR, Fisher PA (2006) Bringing basic research on early experience and stress neurobiology to bear on preventive interventions for neglected and maltreated children. Development and Psychopathology 18: 651–677. 17152395

[56] Caplan RD, Cobb S, French JRP (1979) White-collar work load and cortisol - disruption of a circadian-rhythm by job stress. Journal of Psychosomatic Research 23: 181–192. 573796 10.1016/0022-3999(79)90003-5

[57] Heim C, Ehlert U, Hellhammer DH (2000) The potential role of hypocortisolism in the pathophysiology of stress-related bodily disorders. Psychoneuroendocrinology 25: 1–35. 10633533 10.1016/s0306-4530(99)00035-9

[58] Dowd JB, Simanek AM, Aiello AE (2009) Socio-economic status, cortisol and allostatic load: A review of the literature. International Journal of Epidemiology 38: 1297–1309. 19720725 10.1093/ije/dyp277 PMC2755130

[59] Armstead CA, Anderson NB, Adams-Campbell LL, Hebert JR, Muna WFT (2010) Urbanicity affects blood pressure and heart rate reactivity to a speech stressor in Cameroon. Ethnicity & Disease 20: 251–256. 20828098 PMC3773215

[60] Diamond LM, Fagundes CP, Cribbet MR (2012) Individual Differences in Adolescents' Sympathetic and Parasympathetic Functioning Moderate Associations Between Family Environment and Psychosocial Adjustment. Developmental Psychology 48: 918–931. 22268602 10.1037/a0026901

[61] Ellenbogen MA, Hodgins S (2009) Structure provided by parents in middle childhood predicts cortisol reactivity in adolescence among the offspring of parents with bipolar disorder and controls. Psychoneuroendocrinology 34: 773–785. 19193493 10.1016/j.psyneuen.2008.12.011

[62] Engert V, Efanov SI, Dedovic K, Duchesne A, Dagher A, et al (2010) Perceived early-life maternal care and the cortisol response to repeated psychosocial stress. Journal of Psychiatry & Neuroscience 35: 370–377. 20964960 10.1503/jpn.100022 PMC2964367

[63] Hellhammer DH, Wade S (1993) Endocrine correlates of stress vulnerability. Psychotherapy and Psychosomatics 60: 8–17. 8234641 10.1159/000288675

[64] Liang SW, Jemerin JM, Tschann JM, Irwin CE, Wara DW, et al (1995) Life events, cardiovascular reactivity, and risk behavior in adolescent boys. Pediatrics 96: 1101–1105. 7491228

[65] Elzinga BM, Roelofs K, Tollenaar MS, Bakvis P, van Pelt J, et al (2008) Diminished cortisol responses to psychosocial stress associated with lifetime adverse events - A study among healthy young subjects. Psychoneuroendocrinology 33: 227–237. 18096322 10.1016/j.psyneuen.2007.11.004

[66] Lovallo WR, Farag NH, Sorocco KH, Cohoon AJ, Vincent AS (2012) Lifetime adversity leads to blunted stress axis reactivity: Studies from the Oklahoma Family Health Patterns Project. Biological Psychiatry 71: 344–349. 22112928 10.1016/j.biopsych.2011.10.018 PMC3264696

[67] Prince van Leeuwen A, Creemers HE, Greaves-Lord K, Verhulst FC, Ormel J, et al (2011) Hypothalamic-pituitary-adrenal axis reactivity to social stress and adolescent cannabis use: the TRAILS study. Addiction (Abingdon, England) 106: 1484–1492. 10.1111/j.1360-0443.2011.03448.x 21631618

[68] Tick NT, van der Ende J, Verhulst FC (2007) Twenty-year trends in emotional and behavioral problems in Dutch children in a changing society. Acta Psychiatrica Scandinavica 116: 473–482. 17997726 10.1111/j.1600-0447.2007.01068.x

[69] Dieleman GC, van der Ende J, Verhulst FC, Huizink AC (2010) Perceived and physiological arousal during a stress task: Can they differentiate between anxiety and depression? Psychoneuroendocrinology 35: 1223–1234. 20219286 10.1016/j.psyneuen.2010.02.012

[70] Evans BE, Greaves-Lord K, Euser AS, Franken IHA, Huizink AC (2012) The relation between hypothalamic–pituitary–adrenal (HPA) axis activity and age of onset of alcohol use. Addiction 107: 312–322. 21752143 10.1111/j.1360-0443.2011.03568.x

[71] Sapolsky RM, Romero LM, Munck AU (2000) How do glucocorticoids influence stress responses? Integrating permissive, suppressive, stimulatory, and preparative actions. Endocrine Reviews 21: 55–89. 10696570 10.1210/edrv.21.1.0389

[72] Aardal E, Holm AC (1995) Cortisol in saliva - Reference ranges and relation to cortisol in serum. European Journal of Clinical Chemistry and Clinical Biochemistry 33: 927–932. 8845424 10.1515/cclm.1995.33.12.927

[73] Mulder LJM, Van Dellen HJ, Van der Meulen P, Opheikens B (1988) CARSPAN: a spectral analysis program for cardiovascular time series. In: Maarse FJ, Mulder LJM, Akkerman A, editors. Computers in psychology: methods, instrumentation and psychodiagnostics. Lisse: Swets and Zeitlinger. pp. 39–47.

[74] Van Steenis HG, Tulen JHM, Mulder LJM (1994) Heart-rate-variability spectra based on nonequidistant sampling - the spectrum of counts and the instantaneous heart-rate spectrum. Medical Engineering & Physics 16: 355–362. 7952673 10.1016/1350-4533(90)90001-o

[75] Marshall WA, Tanner JM (1970) Variations in the pattern of pubertal changes in boys. Archives of Diseases in Childhood 45: 13–23. 5440182 10.1136/adc.45.239.13 PMC2020414

[76] Buss AHP, Plomin R (1984) Temperament: Early developing personality traits. Hillsdale, New Jersey: Erlbaum.

[77] Lederbogen F, Kirsch P, Haddad L, Streit F, Tost H, et al (2011) City living and urban upbringing affect neural social stress processing in humans. Nature 474: 498–501. 21697947 10.1038/nature10190

[78] Statistics CBf (2011) Statistics neighborhoods 2004–2010. Statline. Den Haag/Heerlen: Central Bureau for Statistics.

[79] Statistics CBo (2010) Standard Classification of Professions. In: Statistics CBf, editor. the Netherlands.

[80] Perris C, Jacobsson L, Lindstrom H, Knorring LV, Perris H (1980) Development of a new inventory for assessing memories of parental rearing behavior. Acta Psychiatrica Scandinavica 61: 265–274. 7446184 10.1111/j.1600-0447.1980.tb00581.x

[81] Markus MT, Lindhout IE, Boer F, Hoogendijk THG, Arrindell WA (2003) Factors of perceived parental rearing styles: The EMBU-C examined in a sample of Dutch primary school children. Personality and Individual Differences 34: 503–519.

[82] Frick PJ (1991) The Alabama Parenting Questionnaire: Unpublished rating scale. New Orleans: University of New Orleans: Department of Psychology.

[83] Amone-P'Olak K, Ormel J, Huisman M, Verhulst FC, Oldehinkel AJ, et al (2009) Life stressors as mediators of the relation between socioeconomic position and mental health problems in early adolescence: The TRAILS study. Journal of the American Academy of Child & Adolescent Psychiatry 48: 1031–1038. 19707163 10.1097/CHI.0b013e3181b39595

[84] Korhonen T, Huizink AC, Dick DM, Pulkkinen L, Rose RJ, et al (2008) Role of individual, peer and family factors in the use of cannabis and other illicit drugs: A longitudinal analysis among Finnish adolescent twins. Drug and Alcohol Dependence 97: 33–43. 18455885 10.1016/j.drugalcdep.2008.03.015 PMC2574687

[85] Westenberg PM, Bokhorst CL, Miers AC, Sumter SR, Kallen VL, et al (2009) A prepared speech in front of a pre-recorded audience: Subjective, physiological, and neuroendocrine responses to the Leiden Public Speaking Task. Biological Psychology 82: 116–124. 19576261 10.1016/j.biopsycho.2009.06.005

[86] Gaab J, Rohleder N, Nater UM, Ehlert U (2005) Psychological determinants of the cortisol stress response: the role of anticipatory cognitive appraisal. Psychoneuroendocrinology 30: 599–610. 15808930 10.1016/j.psyneuen.2005.02.001

[87] Davidson MC, Amso D, Anderson LC, Diamond A (2006) Development of cognitive control and executive functions from 4 to 13 years: Evidence from manipulations of memory, inhibition, and task switching. Neuropsychologia 44: 2037–2078. 16580701 10.1016/j.neuropsychologia.2006.02.006 PMC1513793

[88] Davis C (1988) Reliability of psychophysiological assessment within temperament groups. International Journal of Psychophysiology 6: 299–305. 3225206 10.1016/0167-8760(88)90017-7

[89] Kagan J, Reznick JS, Snidman N (1988) Biological bases of childhood shyness. Science 240: 167–171. 3353713 10.1126/science.3353713

[90] Tyrka AR, Wier LM, Anderson GM, Wilkinson CW, Price LH, et al (2007) Temperament and response to the Trier Social Stress Test. Acta Psychiatrica Scandinavica 115: 395–402. 17430418 10.1111/j.1600-0447.2006.00941.x PMC4469468

[91] Tarullo AR, Gunnar MR (2006) Child maltreatment and the developing HPA axis. Hormones and Behavior 50: 632–639. 16876168 10.1016/j.yhbeh.2006.06.010

[92] Lupien SJ, McEwen BS, Gunnar MR, Heim C (2009) Effects of stress throughout the lifespan on the brain, behaviour and cognition. Nature Reviews Neuroscience 10: 434–445. 19401723 10.1038/nrn2639

[93] Huizink AC, Ferdinand RF, Ormel J, Verhulst FC (2006) Hypothalamic-pituitary-adrenal axis activity and early onset of cannabis use. Addiction 101: 1581–1588. 17034437 10.1111/j.1360-0443.2006.01570.x

[94] Evans BE, Greaves-Lord K, Euser AS, Tulen JHM, Franken IHA, et al (2012) Alcohol and tobacco use and heart rate reactivity to a psychosocial stressor in an adolescent population. Drug and Alcohol Dependence 126: 296–303. 22726913 10.1016/j.drugalcdep.2012.05.031

[95] Linden W, Earle TL, Gerin W, Christenfeld N (1997) Physiological stress reactivity and recovery: Conceptual siblings separated at birth? Journal of Psychosomatic Research 42: 117–135. 9076640 10.1016/s0022-3999(96)00240-1

